# Differences in the starvation-induced autophagy response in MDA-MB-231 and MCF-7 breast cancer cells

**DOI:** 10.1080/19768354.2017.1330763

**Published:** 2017-05-30

**Authors:** Wanyun Zhu, Hao Qu, Kaixiang Xu, Baoyu Jia, Haifeng Li, Yimin Du, Guangming Liu, Hong-Jiang Wei, Hong-Ye Zhao

**Affiliations:** a College of Pharmacy and Chemistry, Dali University, Dali, People’s Republic of China; b State Key Laboratory for Conservation and Utilization of Bio-Resources in Yunnan, Yunnan Agricultural University, Kunming, People’s Republic of China; c Key Laboratory of Agricultural Biodiversity and Plant Disease Management of China Education Ministry, Yunnan Agricultural University, Kunming, People’s Republic of China; d College of Plant Protection, Yunnan Agricultural University, Kunming, People’s Republic of China; e Key Laboratory of Animal Nutrition and Feed of Yunnan Province, Yunnan Agricultural University, Kunming, People’s Republic of China

**Keywords:** MDA-MB-231, MCF-7, starvation, autophagy-related genes, autophagy signaling pathway-related genes

## Abstract

Breast cancer is a heterogeneous disease with distinct subtypes that have made targeted therapy of breast cancer challenging. Previous studies have demonstrated that an altered autophagy capacity can influence the development of breast cancer. However, the molecular differences in starvation-induced autophagic responses in MDA-MB-231 and MCF-7 cells have not been fully elucidated. In this study, we found that an increase of LC3B-II protein expression level and a decrease of the p62 protein expression level in both cells treated by Earle’s balanced salt solution. Meanwhile, we observed an increase of autophagosome using transmission electron microscopy and an enhancement in the green fluorescence intensity of LC3B protein by confocal microscopy. Furthermore, we detected the expression of 13 autophagy-related (*ATG*) genes and 11 autophagy signaling pathway-related genes using qPCR. Among 13 *ATG* genes, we found that 6 genes were up-regulated in treated MDA-MB-231 cells, while 4 genes were up-regulated and 1 gene was down-regulated in treated MCF-7 cells. In addition, among 11 autophagy signaling pathway-related genes, 7 genes were up-regulated in treated MDA-MB-231 cells, while 5 genes were up-regulated and 1 gene was down-regulated in treated MCF-7 cells. These findings suggest that the autophagic response to starvation was different in the two treated cell lines, which will contribute to further study on the molecular mechanism of starvation-induced autophagy and improve the targeted therapy of breast cancer.

## 1. Introduction

Breast cancer is the most common type of cancer in women and the second leading cause of cancer death in the world (Ferlay et al. 2015). It is a heterogeneous disease with distinct molecular profiles and clinical properties. Estrogen receptor (ER)-positive and ER-negative breast cancers are the two most distinctive subtypes, and MCF-7 and MDA-MB-231 cell lines, respectively, are typical representatives of the two subtypes (Zardavas et al. 2015). It has been reported that the both cell lines have different prognoses and drug responses (Marinello et al. 2016). Clinically, despite the development of several treatment strategies for breast cancer, an increase in the death rate for breast cancer seems to be inevitable (Tang et al. 2016). Therefore, more efficient adjuvant targeted therapeutic strategies need be studied.

Autophagy is a dramatic protein degradation process in which superfluous or damaged organelles and proteins are sequestered, delivered to lysosomes and then digested, leading to the maintenance of cellular homeostasis (de Duve 1983). Inversely, deregulation of autophagy has been linked to several organismal pathologies including cancer (Levine & Kroemer 2008; Mowers et al. 2017). Recently, it has been shown that autophagy plays a pivotal role in growth, metastasis and invasion of breast cancer (Zhou et al. 2016), and the function of autophagy is distinct in different breast cancer subtypes, for example, cytoprotection in MDA-MB-231 cells and cytotoxicity in MCF-7 cells has been observed during gemcitabine-induced autophagy (Shen et al. 2016). Therefore, the adaptive regulation of autophagy could augment the effects of anti-cancer therapy in breast cancer (Rebecca & Amaravadi 2016).

Autophagy can be activated in response to stress stimuli including starvation. Starvation is the most potent known physiological inducer of autophagy, and it has been commonly used to study the molecular mechanism of autophagy (Mizushima et al. 2010). Upon starvation, the core process of autophagy in mammalian cells is regulated by autophagy-related (*ATG*) genes, which are categorized into five groups: (i) the ULK complex that initiates autophagosome formation (Mizushima 2010); (ii) the Beclin-1/PI3K complex for nucleation (Funderburk et al. 2010); (iii–iv) the two ubiquitylation-like systems ATG8/LC3 and ATG12-ATG5 that regulate elongation and closure of autophagosomes (Xu et al. 2015); and (v) ATG9 and its cycling system, which supply the lipid for the expanding membrane (Yamamoto et al. 2012). It has been indicated that the inhibition of some *ATG* genes has an impact on tumor progression in starvation-induced autophagy (Gu et al. 2014; Wei et al. 2015). Furthermore, impaired function of some *ATG* genes is related to different types of cancer (Amaravadi et al. 2016). Therefore, a comprehensive understanding of the molecular mechanism differences in autophagic responses during starvation in tumor cells is necessary.

Some studies have demonstrated that anti-cancer drugs induce autophagy through multiple mechanisms in MDA-MB-231 and MCF-7 cells (Zarzynska 2014). However, the molecular differences in the autophagic response during starvation in these breast cancer cells have not been fully elucidated. In this study, we investigated the molecular differences in starvation-induced autophagy in MDA-MB-231 and MCF-7 breast cancer cells.

## 2. Materials and methods

### 2.1. Reagents and antibodies

Earle’s balanced salt solution (EBSS) was purchased from Sigma-Aldrich (St. Louis, MO, USA). Antibodies against LC3B and p62/SQSTM1 were obtained from Sigma-Aldrich (L7543 and P0067), and β-actin antibody was purchased from Sigma-Aldrich (A5441).

### 2.2. Cell lines and cell cultures

The human breast cancer cell lines MDA-MB-231 and MCF-7 were purchased from the Shanghai Institute Cell Bank and were cultured in basic (1 ×) Dulbecco's modified Eagle's medium (Gibco, NY, USA) supplemented with 10% heat-inactivated fetal bovine serum (Gibco) and 100 IU/ml penicillin. The cells were seeded in gelatin-coated 75-cm^2^ flasks and cultured in 10 ml of medium at 37°C in a humidified atmosphere of 5% CO_2_ in air.

### 2.3. Protein extraction and immunoblotting

After MDA-MB-231 and MCF-7 cells were treated with EBSS at 2, 4, 6 and 8 h, cells were washed twice with phosphate buffered saline (PBS) and collected. Then, the total protein concentration of cell lysates was determined using a BCA protein assay kit (Beyotime, Shanghai, China). Protein samples (total protein: 20 μg) were separated by 12% sodium dodecyl sulfate-polyacrylamide gel electrophoresis and transferred onto a poly vinylidene fluoride membrane. The membranes were incubated for 60 min in 5% bovine serum albumin (BSA) buffer (Solarbio, Beijing, China) with gentle shaking to block non-specific binding before incubation with the diluted primary antibody (LC3B: 1:1000, p62: 1:1000) overnight at 4°C. Subsequently, the membranes were incubated with the 5000-fold diluted secondary antibody (Santa Cruz, CA, USA) for 90 min at room temperature. The membrane was washed three times in PBS, for 10 min each time, and the membrane was incubated for 3 min with a chemiluminescence (ECL) reagent (Easysee Western Blot Kit, Transgene, Alsace, France). Finally, the membranes were exposed in an imaging system (Bio-Rad, Hercules, USA).

### 2.4. Transmission electron microscopy (TEM)

Samples used for TEM analysis were harvested and washed twice with cold PBS (pH 7.4). Before being dehydrated in ethanol, samples were fixed in 2.5% glutaraldehyde for 30 min at room temperature and incubated overnight at 4°C. Samples were washed three times with 0.1 M phosphoric acid buffer solution and were post-fixed with 1% osmium tetroxide for 2–3 h at 4°C. Then, samples were infiltrated with a mixed solution of acetone and embedding solution and embedded in Spurr’s resin for the preparation of ultrathin sections. After staining with 3% uranyl acetate and lead citrate, ultrathin sections were examined using a transmission electron microscope (JEM 1011; JEOL).

### 2.5. Confocal microscopy

MDA-MB-231 and MCF-7 cells were seeded onto 24-chamber culture slides and treated with EBSS at 4 and 8 h, respectively. After fixation in methanol for 10 min and blocking with a buffer containing 1% BSA and 0.1% Triton X-100 for 1 h, cells were incubated with a primary antibody against LC3B from Sigma-Aldrich (L7543) diluted to 1:200 with PBS containing 1% BSA at 4°C overnight. Cells were then incubated for 1 h with 1:400 secondary fluorescence-conjugated antibodies to visualize the binding sites of the primary antibody with laser confocal microscopy (OLYMPUS FV 1000, Tokyo, Japan).

### 2.6. Quantitative real-time polymerase chain reaction (q-PCR) assays

MDA-MB-231 and MCF-7 cells were treated with EBSS at 4 and 8 h. Total RNA was isolated using TRIpure reagent (BioTeke, China) according to the manufacturer’s instructions. cDNA was synthesized from total RNA using a PrimeScript RT reagent kit (TaKaRa, Japan). The obtained cDNA was used as a template in SYBR green-based q-PCR (CFX-96, Bio-Rad, Hercules, CA, USA). The mRNA expression levels of the *ATG* genes were assessed with quantitative polymerase chain reaction (q-PCR). GAPDH was used for normalization. The primers are shown in Table 1.

**Table 1.** The primers for q-PCR.

Gene	Primers sequence (5′ to 3′)	
SESN1	F:CCCCTACATTATCGTCACTACA	R:CAAGGTCTATGGGCTAACACT
PTEN	F:TTGACCAATGGCTAAGTGAAG	R:AATACCTCCTGTAGGATCTGC
VPS34	F:GTACAACGCGAAAGTGGAAAT	R:AACAACTGTGCAGGCATAAGG
MTOR	F:TTACCGCTGAGTACGTGGAATT	R:AATGTTGTCAAAGAAGGGTTGC
EPG5	F:GCTTTTGAACTACTCACGATG	R:ACTCTTTGGTAGCGATGATAG
TSC1	F:AAATTCCACCTCCGACGAGA	R:TCAGTCTGTCCAGCACTTCCA
VPS15	F:TGTTACTTTGCTAAGTGACCCTG	R:CACGTAGATGCCAATCATTCTT
AKT	F:CGGCAAGGTGATCCTGGTGAA	R:CGGTCGTGGGTCTGGAAAGAGTA
LKB1	F:ACATCTGGTCGGCTGGGGTCA	R:CGGTTCGTACTCAAGCATCCCTTT
FOXO1	F:ATGGCAGCCAGGCATCTCATA	R:CTTGGGTCAGGCGGTTCATAC
BECN1	F:TGAGGGATGGAAGGGTCTAAG	R:CCTGGGCTGTGGTAAGTAATG
LMNA	F:TAGACCCTGGGTGGGCTCTGTG	R:GGAGGCAAGGGCTCTTTAGCG
ATG13	F:CGGCTACCTGCGGTTCATCTC	R:CGCTACAGTACGGACGCCCTATT
ULK1	F:TGGCTAATTCTGTCTACCTGGTTATGG	R:CGGTGGATGATGCCTTTGCTG
ATG2A	F:CCCGTTGCCAATCTGCTGTGA	R:AGGGTCCAAAAGCCTCCGTCC
ATG16L1	F:CACTAATATCTTTGGGAGACGCTCTG	R:AACCGGGAACCTGGACTGAAC
DRAM1	F:CAGGGTACTGTTTATTTGCTCCTT	R:CATTTCAAGGGCTGCTTCTTC
ATG7	F:CAAGGTCAAAGGACGAAGATAACA	R:TACGGTCACGGAAGCAAACAA
ATG14	F:CTCGGTGACCTCCTGGTTTAA	R:TAGAAATGTTTGTCTCCCTGCTTA
AMBRA1	F:TAATGTGAATAGAGGAACAAGTGGGTAT	R:AACAGGTGGACAGGGCAAAGC
ATG10	F:TACCCTTGGATGATTGTGAAG	R:AGCAGTCGCATCTTATAGCAC
ATG4A	F:ATGGCACAAATGGGTGTAGGA	R:TGTCAGCACTCAAGGGAAGGA
ATG5	F:CAGAATGCAGGGAACACTAAG	R:GATGCTGGTACAATAATGAATGAG
ATG9A	F:TACCTCCGCCACTTCAACGAG	R:CGTCATAAATGGTGAGGGCAATA
GAPDH	F:GGCGCTGAGTACGTCGTGGAGT	R:AGTTGGTGGTGCAGGAGGCATT

### 2.7. Statistical analysis

Statistical comparisons were performed using Student’s *t*-test. Quantitative data are expressed as the means ± SD. *p < .05 and **p < .01 versus the control were considered significant.

## 3. Results

### 3.1. Starvation induces autophagy in MDA-MB-231 and MCF-7 cells

**Figure 1.**
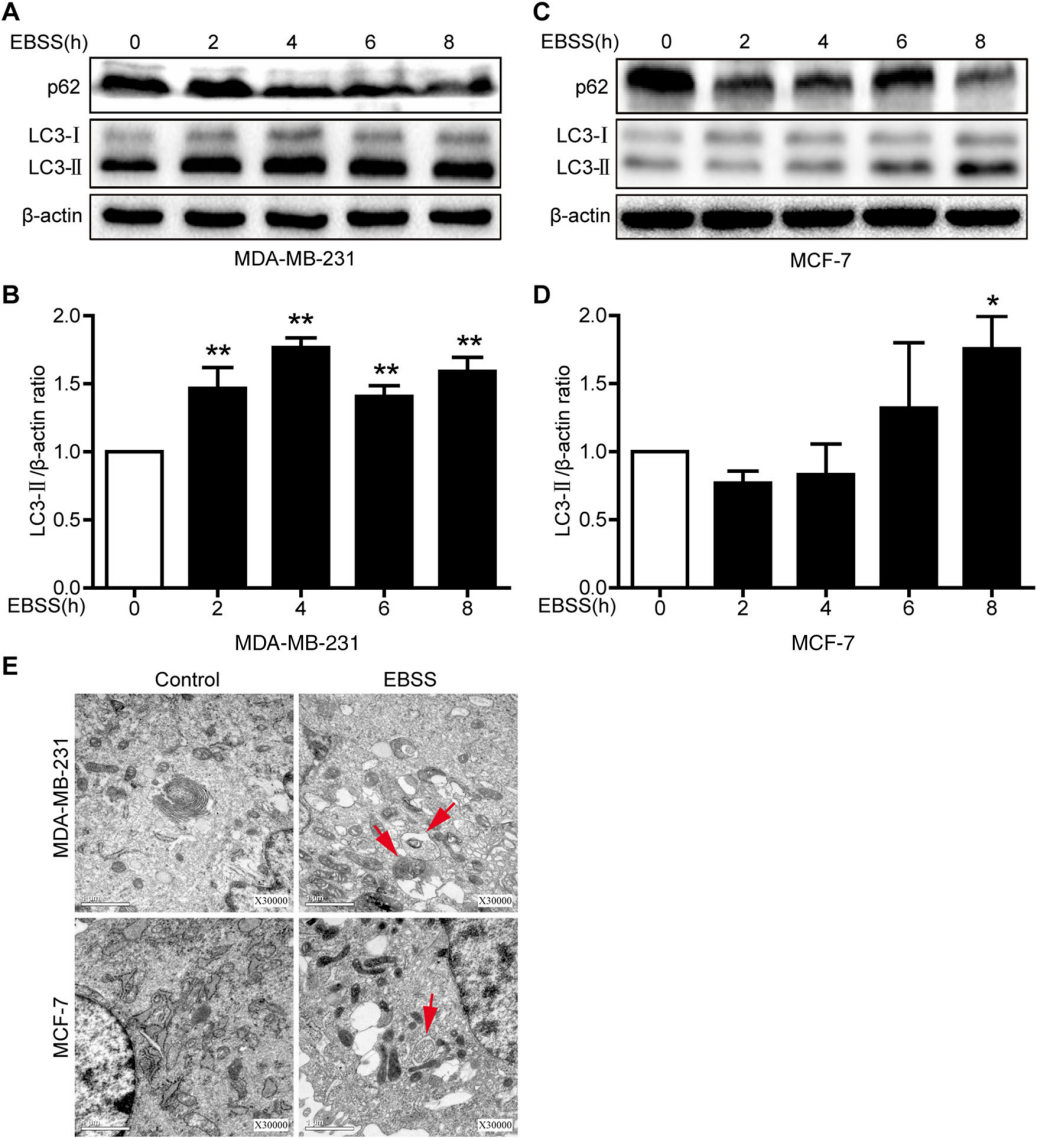
Starvation induces autophagy in MDA-MB-231 and MCF-7 cells. Both cell lines were treated with EBSS for 0 h, 2 h, 4 h, 6 h and 8 h. (a,c) The protein expression levels of LC3B and p62 in the both treated cells were analyzed with immunoblotting. β-actin was used as an internal control. (b,d) Quantification of the LC3-II protein expression level in the both treated cells. *p < .05 and **p < .01 versus the control were considered significant. (e) Representative electron micrographs for both types of cells. MDA-MB-231 and MCF-3 cells were treated with EBSS for 4 h and 8 h, respectively. Untreated cells were used as a control. Red arrows refer to autophagy vacuoles. Scale bars = 1 μm.

LC3B is an important autophagy marker. It is recruited to the autophagosomal membrane, and increased LC3B-II indicates the occurrence of autophagy (Barth et al. 2010). p62/SQSTM1 is a highly conserved scaffolding protein involved in the transportation of ubiquitinated proteins destined for proteasomal degradation, and a decrease in p62 expression indicates autophagy flux (Pankiv et al. 2007). To ensure the peak time of the starvation-induced autophagic response, we treated both breast cancer cell lines with EBSS at 2, 4, 6 and 8 h. We found that the LC3B-II protein expression level was to peak at 4 h and significantly increased 1.76-fold in treated MDA-MB-231 cells, while the LC3B-II protein expression level was to peak at 8 h and significantly increased for 1.75-fold in treated MCF-7 cells. In addition, a decrease in the p62 protein expression level was found in the both cell lines (Figure 1(a–d)). TEM is broadly used as a ‘gold standard’ test for autophagy, and the presence of autophagosomes in TEM images is considered both a hallmark and evidence of autophagy (Mizushima & Komatsu 2011). To further determine whether autophagy was induced in MDA-MB-231 cells treated with EBSS for 4 h and MCF-7 cells treated with EBSS for 8 h. The both cell lines treatment with EBSS was performed by TEM and displayed an increase in autophagosomes compared with controls (Figure 1(e)). These results demonstrated that starvation successfully induced autophagy in MDA-MB-231 and MCF-7 cells, and the peak time of the autophagic response was 4 and 8 h, respectively.

### 3.2. Effect of starvation on the expression level of the autophagosomal marker LC3B in MDA-MB-231 and MCF-7 cells

**Figure 2.**
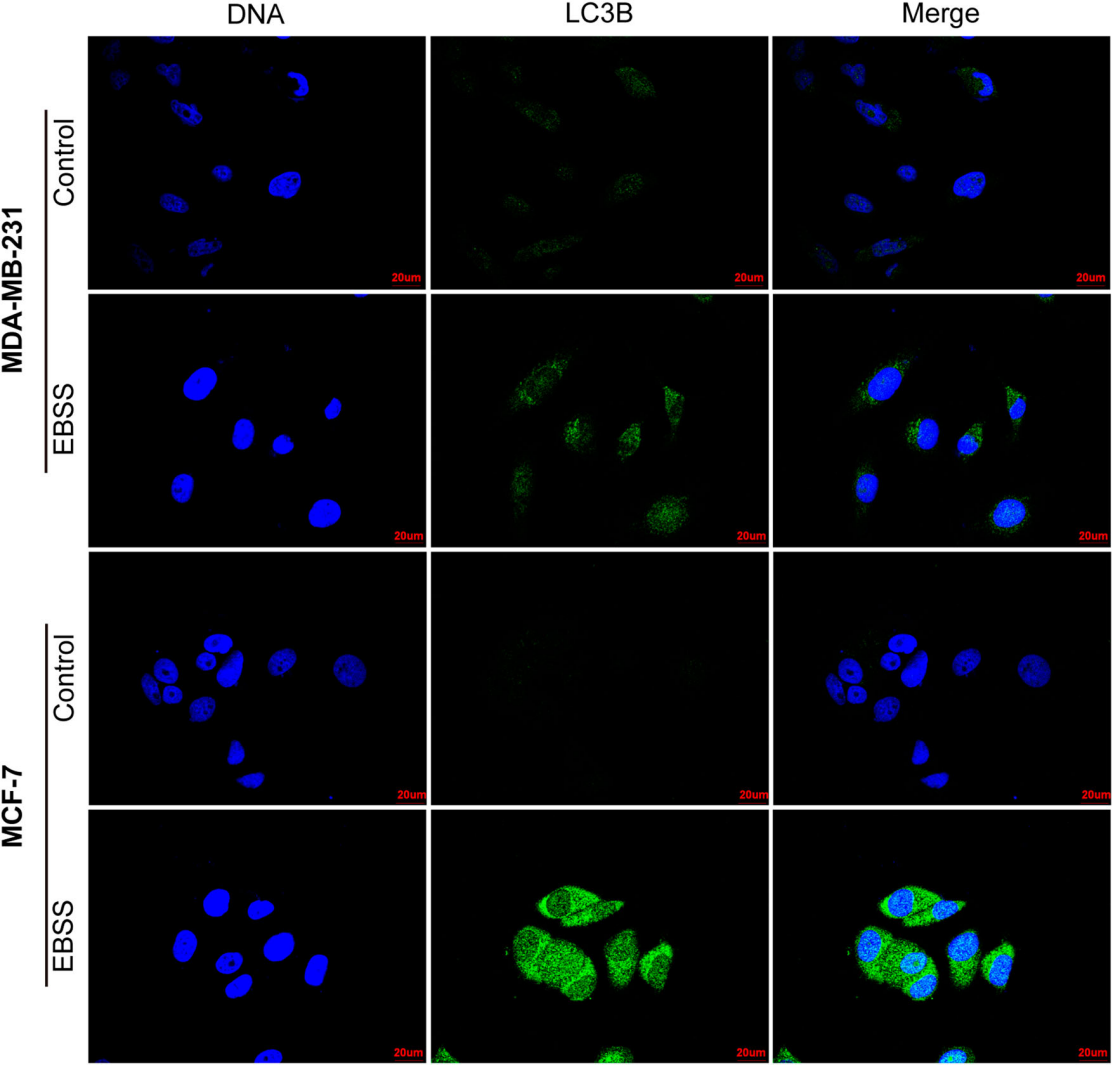
Confocal microscopy images of LC3B in MDA-MB-231 and MCF-7 cells. MDA-MB-231 cells were treated with EBSS for 4 h and MCF-7 cells for 8 h, and were analyzed with confocal microscopy. Cells were stained with antibodies against LC3B (green), nuclei were stained blue with Hoechst 33342. Scale bars = 20 μm.

To further ensure that starvation-induced autophagy in MDA-MB-231 and MCF-7 cells, we treated the both cell lines with EBSS for 4 or 8 h and detected the expression level of the autophagosomal marker LC3B using confocal microscopy. We observed that an enhancement in the green fluorescence intensity of autophagosomal marker LC3B protein in both treated cell lines compared with those of control (Figure 2). The results further confirmed that starvation-induced autophagy in MDA-MB-231 and MCF-7 cells at the indicated times.

### 3.3. Effect of starvation on the ATG genes mRNA expression level in MDA-MB-231 and MCF-7 cells

**Figure 3.**
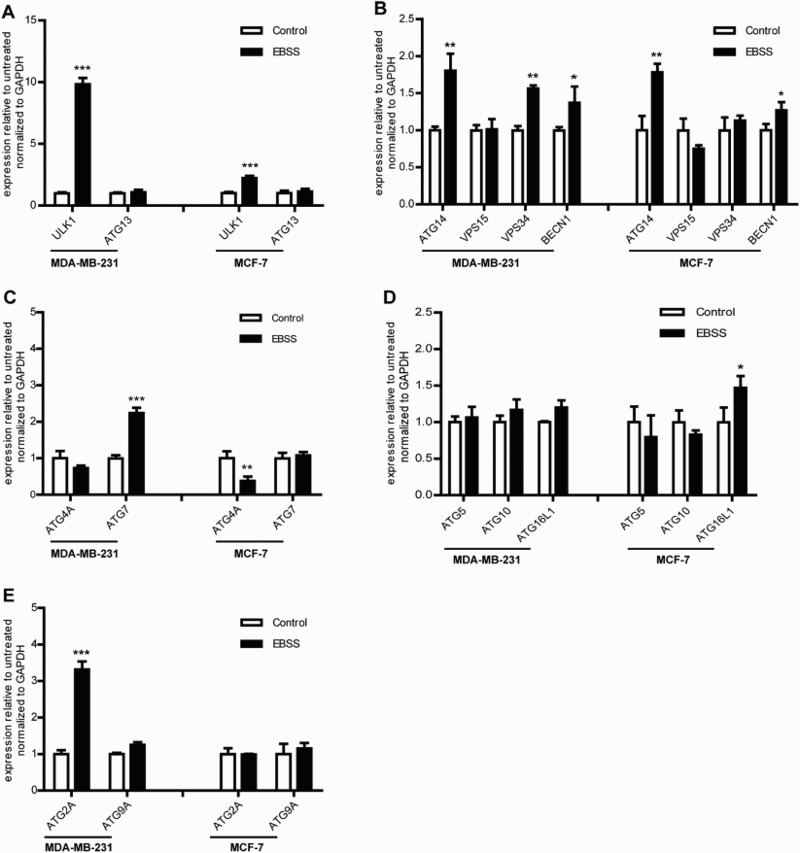
Effect of starvation on the *ATG* genes mRNA expression level in MDA-MB-231 and MCF-7 cells. The cells were treated with as described above. Untreated cells were used as a control. (a) The mRNA expression levels of the autophagosome initiation-related genes *ULK1* and *ATG13* in the both treated cell lines. (b) The mRNA expression levels of the nucleation-related genes *ATG14*, *VPS15*, *VPS34* and *BECN1* in the both treated cell lines. (c) The mRNA expression levels of the elongation-related genes *ATG4A* and *ATG7* in the both treated cell lines. (d) The mRNA expression levels of the elongation-related genes *ATG5*, *ATG10* and *ATG16L1* in the both treated cell lines. (e) The mRNA expression levels of the degradation cycling-related genes *ATG2A* and *ATG9A* in the both treated cell lines. *p < .05 and **p < .01 versus the control were considered significant.

The formation process of autophagosome was regulated by *ATG* genes, then we detected the *ATG* genes mRNA expression level during starvation using qPCR. We found that the similarities and differences of *ATG* genes response to starvation in both treated cell lines as shown in Figure 3, and the mRNA expression levels of the *ULK1*, *ATG14* and *BECN1* genes were significantly up-regulated and *ATG5*, *ATG9A*, *ATG10*, *ATG13* and *VPS15* genes had no change in the both treated cells. The differences were that *VPS34*, *ATG7* and *ATG2A* genes were significantly up-regulated in treated MDA-MB-231 cells compared with those of the control, whereas had no change in MCF-7 cells (Figure 3(b,c,e)). *ATG4A* gene was down-regulated and *ATG16L1* gene was significantly up-regulated in treated MCF-7 cells, whereas had no change in MDA-MB-231 cells (Figure 3(c,d)). These results demonstrated that the *ATG* gene response to starvation in the process of autophagosome formation was distinctly different in both cell lines.

### 3.4. Effect of starvation on the mRNA expression level of autophagy signaling pathway-related genes in MDA-MB-231 and MCF-7 cells

**Figure 4.**
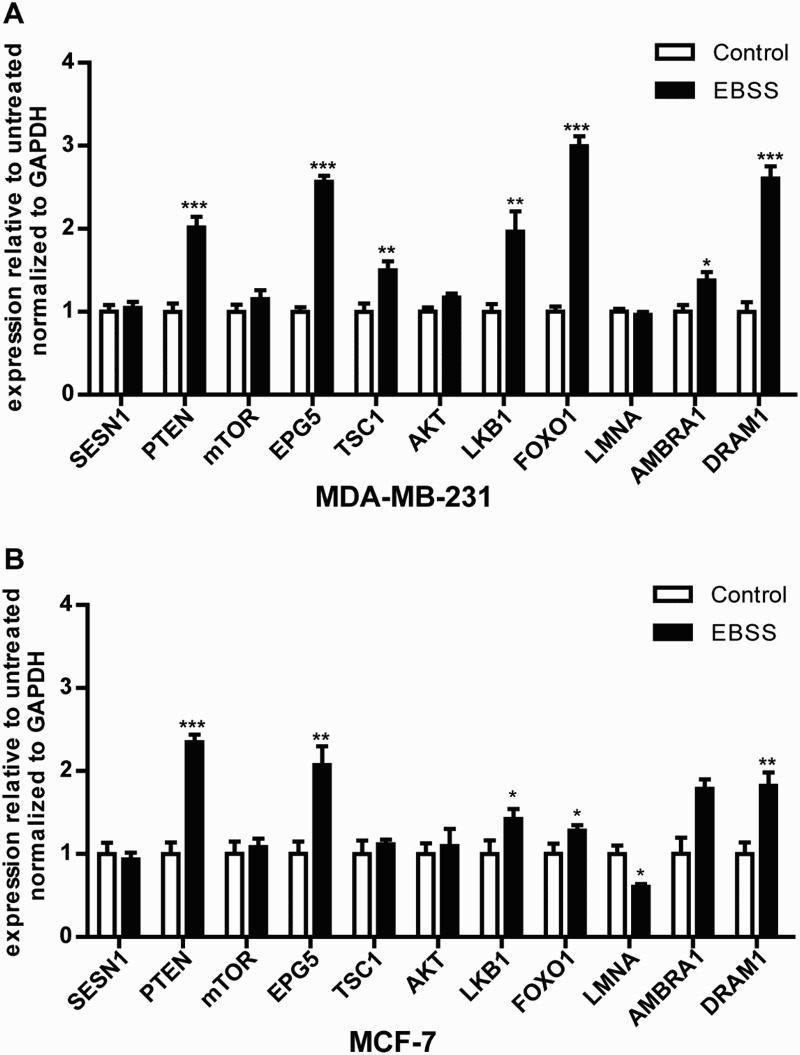
Effect of starvation on the mRNA expression levels of autophagy signaling pathway-related genes in MDA-MB-231 and MCF-7 cells. Both types of cells were treated as described above. (a,b) The mRNA expression levels of the autophagy signaling pathway-related genes *SESN1*, *PTEN*, *mTOR*, *EPG5*, *TSC1*, *AKT*, *LKB1*, *FOXO1*, *LMNA*, *AMBRA1* and *DRAM1* in the both treated cell lines. *p < .05 and **p < .01 versus the control were considered significant.

Several studies have indicated that autophagy is also regulated by upstream autophagy genes, such as the *PTEN*, *FOXO1*, *LKB1*, *mTOR*, *SESN1*, *EPG5*, *TSC1*, *AKT*, *LMNA*, *AMBRA1* and *DRAM1* genes, which are essential for autophagy signaling pathways (Alers et al. 2012). We found that the mRNA expression levels of the autophagy signaling pathway-related genes *PTEN*, *EPG5*, *LKB1*, *FOXO1* and *DRAM1* were up-regulated significantly in the both cell lines. Moreover, the expression of the *AMBRA1* gene was significantly up-regulated in MDA-MB-231 cells (Figure 4(a)), whereas there was no change in MCF-7 cells; the expression of the *LMNA* gene was down-regulated significantly in MCF-7 cells (Figure 4(b)), whereas there was no change in MDA-MB-231 cells. The results demonstrated that a portion of the autophagy signaling pathway response to starvation might be different in the both cell lines.

## 4. Discussion

EBSS-induced starvation is a classic method for autophagy induction (Cui et al. 2015). This method has been applied to the study of autophagy in various cancers (Guo et al. 2014; Huangfu et al. 2016). Consistently, we also used EBSS to successfully induce autophagy (Figures 1 and 2). In addition, we further confirmed EBSS-induced autophagy with acridine orange staining in MDA-MB-231 and MCF-7 cells (data not shown). However, interestingly, the peak time of the autophagic response in MDA-MB-231 cells was earlier than that of the MCF-7 cells (Figure 1(b,d)). It has been reported that a delayed and protracted autophagic response depends on the activation of stress-responsive transcription factors (Amaravadi et al. 2016). Therefore, the intra-tumoral heterogeneity, including stress-responsive transcription factors, in MDA-MB-231 and MCF-7 cells may result in the difference. However, whether the activation of stress-responsive transcription factors have an impact on the peak time of the autophagic response during starvation in the both cell lines requires further study.

It has been reported that *ULK1* up-regulation may be a mode of breast cancer cell survival and tumor progression (Pike et al. 2013). *BECN1* and *ATG14* genes were responsible for the vesicle nucleation stage, and were related with tumor progression (Chen & Debnath 2010). We found that *ULK1*, *ATG14* and *BECN1* genes were up-regulated in both cell lines (Figure 3). These findings suggest that targeting *ULK1*, *ATG14* and *BECN1* genes might affect the both cell lines survival and progression.

In addition, the differences in the response of *ATG* genes in the treated cell types were notable. Our results show that *ATG7*, *ATG2A* and *VPS34* genes were significantly increased in MDA-MB-231 cells but not changed in MCF-7 cells (Figure 3(b,c,e)). Tumor-specific *ATG7* gene deficiency causes tumors to prematurely induce p53, proliferation arrest and cell death, which reduces tumor burden (White 2015). The *ATG2A* gene can increase the number and size of cytoplasmic lipid droplets to provide energy for cancer cells (Pfisterer et al. 2014). The *ATG16L1* and *ATG4A* genes were significantly changed in MCF-7 cells but were not changed in MDA-MB-231 cells (Figure 3(c,d)). Abnormal expression levels of some human *VPS34*, *ATG4A* and *ATG16L1* genes occurs in several types of cancer cells, which may be closely related to tumor progression, tumor suppression and cancer therapy resistance (Chen et al. 2014). The results suggest that the efficacy of treatment might be improved through targeting *ATG7*, *ATG2A* and *VPS34* genes in MDA-MB-231 cells or *ATG4A* and *ATG16L1* in MCF-7 cells. Therefore, the differences response to starvation in *ATG* genes described above likely contribute to the targeted therapy of breast cancer. It has been reported that ER status is relevant to the difference in autophagic response in breast cancer (Shen et al. 2016), however, whether the specific role of these genes depends on ER status in anti-cancer therapy needs further study.

Autophagy is also regulated by the upstream autophagy signaling network. Among them, The PI3K/AKT/FOXO, PI3 K/AKT/mTOR and LKB1/AMPK/mTOR signaling pathways play an important role in autophagy (Alers et al. 2012). We found that the expression of *FOXO1*, *PTEN* and *LKB1* was up-regulated in both treated cells (Figure 4), and the expression of *mTOR* and *AKT* showed no changes because *mTOR* and *AKT* were activated by phosphorylation (He et al. 2012). These findings suggest that starvation treatment might induce autophagy by the same signaling pathway as described above in both treated cells.

It has been reported that *LMNA* mutation activates AKT mTOR signaling and impairs autophagy, which results in cell damage that causes laminopathies (Choi & Worman 2013), and down-regulation of the *LMNA* gene in neuroblastoma promotes tumor progression (Nardella et al. 2015). Additionally, over-expression of the *AMBRA1* gene was found in some tumors (Cianfanelli et al. 2015). Interestingly, we found that *LMNA* was down-regulated in MCF-7 cells but was not changed in MDA-MB-231 cells (Figure 4(a,b)). *AMBRA1* gene was up-regulated in MDA-MB-231 cells, while there was no change in MCF-7 cells (Figure 4(a,b)). These findings suggest that starvation treatment might induce autophagy through the LMNA/AKT/mTOR signaling pathway in the treated MCF-7 cells and the AMBRA1/mTOR pathway in the treated MDA-MB-231 cells. Therefore, these findings suggest that targeting the *LMNA* signaling pathway in MDA-MB-231 cells or the *AMBRA1* signaling pathway in MCF-7 cells might improve the efficacy of breast cancer targeted treatment. Based on these findings, we hypothesize that different autophagy signaling pathways might result in differences in the autophagic response by regulating *ATG* genes during starvation in the two types of breast cancer cells. However, the specific mechanism involved in regulation of *ATG* genes by the autophagy signaling pathway during starvation needs further study.

In summary, we found that an increase of LC3B-II protein expression level and a decrease of the p62 protein expression level, an increase of autophagosomes and an enhancement in the green fluorescence intensity of LC3B protein in the both cells treatment with EBSS. In addition, we found the similarities and differences of *ATG* genes and autophagy signaling pathway-related genes response to starvation. These findings will lay the foundation for further study of the molecular mechanism of starvation-induced autophagy and improvements in the targeted treatment of breast cancer.
